# Structure of mammalian plasma fetuin-B and its mechanism of selective metallopeptidase inhibition

**DOI:** 10.1107/S2052252519001568

**Published:** 2019-02-28

**Authors:** Anna Cuppari, Hagen Körschgen, Dirk Fahrenkamp, Carlo Schmitz, Tibisay Guevara, Konstantin Karmilin, Michael Kuske, Mario Olf, Eileen Dietzel, Irene Yiallouros, Daniele de Sanctis, Theodoros Goulas, Ralf Weiskirchen, Willi Jahnen-Dechent, Julia Floehr, Walter Stoecker, Luca Jovine, F. Xavier Gomis-Rüth

**Affiliations:** a Proteolysis Laboratory, Department of Structural Biology, Molecular Biology Institute of Barcelona, CSIC, Barcelona Science Park, Helix Building, c/o Baldiri Reixac 15-21, E-08028 Barcelona, Catalonia, Spain; b Institute of Molecular Physiology, Cell and Matrix Biology, Johannes Gutenberg-University Mainz, Johann-Joachim-Becher-Weg 7, D-55128 Mainz, Germany; c Department of Biosciences and Nutrition and Center for Innovative Medicine, Karolinska Institutet, Blickagången 16, SE-141 83 Huddinge, Sweden; d Biointerface Laboratory, Helmholtz Institute for Biomedical Engineering, RWTH Aachen University Medical Faculty, Pauwelsstrasse 30, D-52074 Aachen, Germany; e ESRF – The European Synchrotron, 71 Rue Jules Horowitz, F-38000 Grenoble, France; f Institute of Molecular Pathobiochemistry, Experimental Gene Therapy and Clinical Chemistry, RWTH Aachen University Hospital, Pauwelsstrasse 30, D-52074 Aachen, Germany

**Keywords:** mammalian fertilization, sperm–egg fusion, polyspermy, metallopeptidase, protein inhibitor, structure determination, protein structure, X-ray crystallography, enzyme mechanisms, multi-protein complexes

## Abstract

Mammalian fetuin-A and fetuin-B are abundant serum proteins with pleiotropic functions. Fetuin-B is a highly selective and potent inhibitor of metallo­peptidases (MPs) of the astacin family, which includes ovastacin in mammals. By inhibiting ovastacin, fetuin-B is essential for female fertility. The crystal structure of fetuin-B was determined unbound and in complex with archetypal astacin, and it was found that the inhibitor has tandem cystatin-type modules (CY1 and CY2). They are connected by an exposed linker with a rigid, disulfide-linked ‘CPDCP-trunk’, and are followed by a C-terminal region (CTR) with little regular secondary structure. The CPDCP-trunk and a hairpin of CY2 form a bipartite wedge, which slots into the active-site cleft of the MP. These elements occupy the nonprimed and primed sides of the cleft, respectively, but spare the specificity pocket so that the inhibitor is not cleaved. The aspartate in the trunk blocks the catalytic zinc of astacin, while the CY2 hairpin binds through a QWV*X*GP motif. The CY1 module assists in structural integrity and the CTR is not involved in inhibition, as verified by *in vitro* studies using a cohort of mutants and variants. Overall, the inhibition conforms to a novel ‘raised-elephant-trunk’ mechanism for MPs, which is reminiscent of single-domain cystatins that target cysteine peptidases. Over 200 sequences from vertebrates have been annotated as fetuin-B, underpinning its ubiquity and physiological relevance; accordingly, sequences with conserved CPDCP- and QWV*X*GP-derived motifs have been found from mammals to cartilaginous fishes. Thus, the raised-elephant-trunk mechanism is likely to be generally valid for the inhibition of astacins by orthologs of fetuin-B.

## Abstract 2

The co-crystal structure of the metallopeptidase astacin with its specific protein inhibitor fetuin-B reveals a novel mechanism of inhibition.

## 1. Introduction   

Fetuin was discovered in 1944 as the most abundant globulin in fetal calf serum (Pedersen, 1944 ▸). Today, fetuins constitute a broad group of liver-derived plasma and matrix proteins (Lee, 2009 ▸). These include fetuin-A, also known as α_2_-Heremans–Schmid glycoprotein, and fetuin-B, which was discovered as a paralog of fetuin-A in 1999 (Olivier *et al.*, 2000 ▸; Denecke *et al.*, 2003 ▸). Fetuins belong to the cystatin family of proteins (Turk & Bode, 1991 ▸), which matches family I25 of the MEROPS database (https://www.ebi.ac.uk/merops). This family is derived from the archetypal reversible inhibitor specific for cysteine peptidases, the monomeric 116-residue chicken egg-white cystatin (ovocystatin; Bode *et al.*, 1988 ▸; Stubbs *et al.*, 1990 ▸). Among the cystatins, fetuins belong to subfamily I25C, also referred to as type-3 cystatins, which groups glycosylated proteins with two or three cystatin-like repeats (Rawlings & Barrett, 1990 ▸; Lee, 2009 ▸). Fetuins consist of tandem cystatin domains (hereafter referred to as CY1 and CY2) followed by a C-terminal region (hereafter referred to as CTR). Another member of this subfamily is histidine-rich glycoprotein, a two-repeat plasma protein from vertebrates that is involved in the vascular, coagulation and immune systems and is not a peptidase inhibitor (Turk & Bode, 1991 ▸). Yet other members are the kininogens, which are the precursors of the kinin hormones and are initiators of the surface-activated blood-coagulation cascade. They comprise three cystatin modules, the last two of which inhibit cysteine peptidases (Salvesen *et al.*, 1986 ▸). Finally, the subfamily also includes endogenous inhibitors of snake-venom metallopeptidases (MPs) from the ADAM/adamalysin family (Gomis-Rüth, 2013 ▸). These inhibitors contain two cystatin modules and operate through an unknown mechanism.

Fetuin-A serum levels are high throughout life and are even higher during fetal development; fetuin-B is less abundant than fetuin-A (Floehr *et al.*, 2016 ▸, 2017 ▸). Despite their common phylogenetic origin and close chromosomal location within all studied vertebrate genomes, fetuin-A and fetuin-B are chemically different and have separate biological functions. An important role in mineralized matrix metabolism has been demonstrated for fetuin-A, among several other moonlighting functions (Jahnen-Dechent *et al.*, 2011 ▸), while fetuin-B is essential for fertilization (Dietzel *et al.*, 2013 ▸; Stöcker *et al.*, 2014 ▸). It blocks the proteolytic activity of ovastacin, a member of the astacin family (Gomis-Rüth, Trillo-Muyo *et al.*, 2012 ▸) within the metzincin clan of MPs (Gomis-Rüth, 2009 ▸; Cerdà-Costa & Gomis-Rüth, 2014 ▸). Ovastacin mediates the hardening of the zona pellucida (ZP), a glycoprotein matrix surrounding the oocyte, by cleaving the ZP component ZP2 after gamete fusion (Burkart *et al.*, 2012 ▸) and thus preventing further sperm from penetrating the oocyte. By blocking ovastacin, fetuin-B counteracts premature ZP hardening before fertilization and therefore maintains female fertility by keeping the ZP penetrable to sperm (Körschgen *et al.*, 2017 ▸).

Detailed biochemical analysis revealed that mammalian fetuin-B inhibits its physiological target ovastacin at picomolar concentrations and most other astacins, for example crayfish astacin, zebrafish nephrosin and human meprins α and β, at nanomolar concentrations (Karmilin *et al.*, 2019 ▸). As no other peptidase type was inhibited, fetuin-B is considered to be an endogenous and specific inhibitor of non-BMP-1-like astacins (Karmilin *et al.*, 2019 ▸). It complements the broad-spectrum pan-peptidase inhibitor α_2_-macroglobulin (Goulas *et al.*, 2017 ▸) and *Xenopus laevis* Sizzled/Ogon, which blocks BMP-1-like astacins (Lee *et al.*, 2006 ▸). In addition, a carp ortholog of fetuin strongly inhibited carp nephrosin, an endogenous astacin family member (Tsai *et al.*, 2004 ▸). Finally, unlike fetuin-B and despite conflicting reports (Yamamoto & Sinohara, 1993 ▸; Yoshida *et al.*, 1996 ▸; Hedrich *et al.*, 2010 ▸), fetuin-A is not a peptidase inhibitor (Lee, 2009 ▸; Dietzel *et al.*, 2013 ▸; Karmilin *et al.*, 2019 ▸; Galembeck & Cann, 1974 ▸): depending on the analyzed peptidase, the apparent inhibitory activity of fetuin-A was owing to contaminating fetuin-B or to chromogenic substrate competition by fetuin-A.

Currently, more than 200 sequences, exclusively from vertebrates, have been annotated as fetuin-B in the UniProt database. This supports a ubiquity and physiological relevance of this protein that is probably associated with the selective and pleiotropic inhibition of a family of MPs. To understand this function at the molecular level and to guide the rational design of small-molecule regulators, we solved the structure of mouse fetuin-B both unbound and in complex with the archetypal MP astacin, which is a valid model for the physiological target ovastacin. We verified the findings using inhibitory assays of recombinant protein variants, and further discuss the implications of this novel inhibition mechanism.

## 2. Materials and methods   

### 2.1. Protein production and purification   

Crayfish astacin was purified from the digestive fluid of the European freshwater crayfish *Astacus astacus* L. as described by Gomis-Rüth *et al.* (1993 ▸). Recombinant mouse pro-ovastacin was obtained as reported by Dietzel *et al.* (2013 ▸) and was proteolytically activated with plasmin (Karmilin *et al.*, 2019 ▸). Human meprin β was obtained as described by Becker *et al.* (2003 ▸) and was activated with trypsin (Fridrich *et al.*, 2016 ▸). Mouse fetuin-A and mouse fetuin-B, as well as the mouse fetuin-B loop-swap variant mFB_H1-swap_, in which the sequence Q^199^WV­SGP^204^ is replaced by Q^78^EDMGP^82^, were hexahistidine-tagged at the C-terminus, cloned in pFASTBac1 vector and expressed in baculovirus-transduced High Five cells as described for meprin (Becker *et al.*, 2003 ▸; Becker-Pauly *et al.*, 2007 ▸). Bovine and human fetuin-B orthologs, as well as the CY2 (residues K^147^–F^265^; mouse fetuin-B residue numbers are shown as superscripts; see UniProt Q9QXC1) and CTR (residues F^266^–P^388^) domains of mouse fetuin-B and point mutants (P^155^A, D^156^A, P^155^A+D^156^A, C^154^S+C^157^S and C^39^S+C^374^S) of mouse fetuin-B, were expressed in ExpiCHO-S cells (ThermoFisher Scientific, Waltham, USA) according to the manufacturer’s specifications (Karmilin *et al.*, 2019 ▸). For the generation of point mutants of mouse fetuin-B, the original pcDNATM3.4 TOPO_mouseFetuB construct (Karmilin *et al.*, 2019 ▸) was used as a template for site-directed mutagenesis. All variants were generated by the QuikChange Lightning Multi Site-Directed Mutagenesis Kit (Agilent Technologies, Santa Clara, USA) using the standard protocol and specific primers. Following mutagenesis, parental DNA was digested by DpnI. Afterwards, XL10-Gold ultracompetent cells were transformed with the resulting PCR products according to the manufacturer’s instructions. The gene sequences were confirmed by DNA sequencing (Eurofins Genomics, Ebersberg, Germany). The chimeric mutant mFB_ABA_ comprised the CY1 (residues A*19*–P*136*; mouse fetuin-A residue numbers are shown in italics, see UniProt P29699) and CTR (residues P*254*–I*345*) domains of mouse fetuin-A plus an intercalated mouse fetuin-B CY2 domain (residues V^145^–Q^269^) and was obtained from adenovirus-infected COS-7 cells (Karmilin *et al.*, 2019 ▸). All proteins carry a cleavable C-terminal hexa­histidine tag and were purified by nickel–nitrilotriacetic acid (Ni^2+^–NTA) affinity chromatography as described previously (Dietzel *et al.* 2013 ▸; Karmilin *et al.* 2019 ▸). Finally, cyclized peptides including the sequences CPDC (full sequence Ac-VSKRKTHTTCPDCPSPIDL) and CPRC (full sequence Ac-DSAEDVRKLCPRCPLLTPFN) were purchased from JPT Peptide Technologies GmbH, Berlin, Germany.

Mouse fetuin-B was also produced in HEK293S cells, which synthesize proteins carrying endoglycosidase H-sensitive Man_5_GlcNAc_2_ N-glycans (Reeves *et al.*, 2002 ▸). For this, a cDNA encoding full-length mouse fetuin-B (GenScript) was cloned in frame with the 3′ hexahistidine tag-encoding sequence of pHLsec2, a mammalian expression vector derived from pHLsec (Aricescu *et al.*, 2006 ▸). The resulting construct was used for transient transfections using 25 kDa branched PEI (Sigma–Aldrich; Aricescu *et al.*, 2006 ▸). The secreted fetuin-B was purified by Ni^2+^–NTA chromatography, deglycosylated with endoglycosidase H and purified by size-exclusion chromatography essentially as described by Bokhove *et al.* (2016 ▸), except that the deglycosylation step was carried out overnight at 4°C. Finally, the protein was concentrated to 7.5–30 mg ml^−1^ in 150 m*M* sodium chloride, 20 m*M* sodium HEPES pH 7.8.

### 2.2. Crystallization and diffraction data collection   

Crystallization assays of the astacin–fetuin-B complex were performed using the sitting-drop vapor-diffusion method. Reservoir solutions were prepared by a Tecan robot and 100 nl crystallization drops were dispensed onto 96 × 2-well MRC plates (Innovadyne) at the joint IBMB/IRB Automated Crystallography Platform at Barcelona Science Park using a Cartesian MicroSys 4000 XL robot (Genomic Solutions) or a Phoenix nanodrop robot (Art Robbins). Plates were kept in Bruker steady-temperature crystal farms at 4 or 20°C. Crystals were obtained by incubating the mouse inhibitor with a sixfold molar excess of the crayfish peptidase. The best crystals were obtained at 20°C in 0.1 µl:0.1 µl drops with protein solution at a concentration of 6 mg ml^−1^ with 200 m*M* sodium chloride, 10 m*M* Tris–HCl pH 7.8 as the buffer and 0.05 *M* ammonium sulfate, 20%(*w*/*v)* polyethylene glycol 2000, 0.1 *M* sodium acetate pH 4.6 as the reservoir solution. Crystals were cryoprotected by rapid passage through drops containing increasing concentrations of glycerol [up to 10%(*v*/*v*)]. It is noteworthy that properly diffracting crystals appeared after 4–5 days and rapidly lost diffracting power after a further 1–2 weeks. SDS–PAGE and N-terminal Edman degradation of washed and dissolved complex crystals revealed that fetuin-B was cleaved at positions S^296^–S^297^ within the CTR, which did not alter its inhibitory properties (data not shown).

Crystals of intact unbound mouse fetuin-B produced in HEK293S cells were grown using a Mosquito robot (TTP Labtech) by sitting-drop vapor diffusion at room temperature against a mother liquor consisting of 100 m*M* sodium acetate, 25%(*w*/*v*) PEG 4000, 8%(*v*/*v*) isopropanol.

Diffraction data for the astacin–fetuin-B complex crystals were collected at 100 K from liquid-nitrogen flash-cryocooled crystals (Oxford Cryosystems 700 Series Cryostream) using a PILATUS 6M detector (Dectris) on the XALOC beamline (Juanhuix *et al.*, 2014 ▸) of the ALBA synchrotron, Cerdanyola, Catalonia, Spain. Diffraction data were processed with *XDS* (Kabsch, 2010*a* ▸) and *XSCALE* (Kabsch, 2010*b* ▸), and were transformed using *XDSCONV* to a format suitable for the *CCP*4 suite of programs (Winn *et al.*, 2011 ▸). The crystals belonged to space group *P*2_1_2_1_2_1_, contained two peptidase–inhibitor complexes per asymmetric unit (molecules *A*/*B* and *C*/*D*, respectively) and diffracted to 3.1 Å resolution.

Diffraction data for the free fetuin-B crystals were collected at 100 K from liquid-nitrogen flash-cryocooled crystals using a PILATUS 6M-F detector (Dectris) on beamline ID29 (de Sanctis *et al.*, 2012 ▸) at the European Synchrotron Radiation Facility (ESRF), Grenoble, France. Three 360° wedges collected from different positions of a single crystal at 7 keV (1.7712 Å) were first integrated and scaled with *XDS* and then merged with *XSCALE*, resulting in a 31-fold redundant data set. The crystals belonged to space group *P*4_1_2_1_2_1_, contained one inhibitor molecule per asymmetric unit and diffracted to 2.3 Å resolution (see Table 1 ▸ for data-processing statistics).

### 2.3. Structure solution and refinement   

The structure of the astacin–fetuin-B complex was solved by maximum-likelihood-scored molecular replacement using *Phaser* (McCoy *et al.*, 2007 ▸) with the coordinates of the protein part of unbound astacin (PDB entry 1ast; Bode *et al.*, 1992 ▸; Gomis-Rüth *et al.*, 1993 ▸) as a search model. These calculations were followed by automated density modification and model building with the *AutoBuild* protocol of the *PHENIX* suite (Terwilliger *et al.*, 2008 ▸), which included twofold averaging. The resulting Fourier map initiated 12 rounds of manual model building with *Coot* (Emsley *et al.*, 2010 ▸), which alternated with crystallographic refinement with *PHENIX* (Afonine *et al.*, 2012 ▸) and *BUSTER*/*TNT* (Smart *et al.*, 2012 ▸) with the inclusion of translation/libration/screw-rotation refinement and noncrystallographic symmetry restraints. The final refined model was comprised of residues A_1_–H_202_ and A_1_–L_200_ [mature astacin residue numbers are shown in subscript, as in Gomis-Rüth *et al.* (1993 ▸) and Guevara *et al.* (2010 ▸); add 49 for residue numbers in the full-length sequence; see UniProt P07584] from astacin molecules *A* and *C*, respectively, plus a zinc cation each, residues R^29^–P^388^ (except for T^218^–Q^227^, L^246^–L^251^ and P^273^–A^302^) plus S^501^–A^502^ from the C-terminal purification tag from molecule *B* and Q^28^–P^388^ plus S^501^ (except for T^218^–H^228^, H^248^–L^251^, S^268^–A^302^, D^314^–P^326^ and P^354^–G^355^) from molecule *D*. One *N*-acetyl-d-glucosamine (NAG) moiety was attached to N^40^ and N^139^ of molecule *B*, and one and two NAG moieties were linked to N^40^ and N^139^ of molecule *D*, respectively. Three glycerols plus 46 solvent molecules completed the model. Some regions of molecule *D* were only poorly defined in the final Fourier maps and were modeled based on molecule *B* to preserve the overall chain continuity, as this led to lower free *R*-factor values. The satisfactory quality of the final model was assessed with the wwPDB Validation Service (https://www.wwpdb.org/validation; Berman *et al.*, 2003 ▸).

The structure of isolated fetuin-B was independently solved by sulfur SAD using the *CRANK*2 (Pannu *et al.*, 2011 ▸) experimental phasing pipeline of *CCP*4 Cloud’s *jsCoFE* (Krissinel *et al.*, 2018 ▸). Using an initial high-resolution cutoff of 4.5 Å, this procedure located a substructure that included 12 sites corresponding to five disulfide bonds and three methionine residues (mean phasing figures of merit of 0.1084 before density modification and 0.4363 after density modification with Fourier recycling) and produced an initial model consisting of 285 amino acids (*R*_work_ = 30.9%, *R*_free_ = 33.8%). This model was expanded using *AutoBuild* within *PHENIX*. It was then manually rebuilt with *Coot* and refined against the first wedge of diffraction data with *PHENIX*. The final model included residues L^31^–P^388^ (except for the segments T^218^–H^228^, S^244^–V^255^, A^270^–T^295^ and E^316^–S^325^), one NAG attached to N^40^ and N^139^, one acetate and 85 solvent molecules. Structure validation was performed with *MolProbity* (Williams *et al.*, 2018 ▸), *Privateer* (Agirre *et al.*, 2015 ▸) and *pdb-care* (Lütteke & Lieth, 2004 ▸). Data-collection, refinement and validation statistics are reported in Table 1 ▸.

### 2.4. Inhibition assays *in vitro*   

The inhibitory capacities of fetuin variants towards mouse ovastacin, human meprin β and crayfish astacin were determined *in vitro* by means of a fluorogenic enzyme-activity assay monitored using a Varioskan Flash 3001 spectral plate reader equipped with the *Skanit* 2.4.3.RE software (Thermo Scientific, Dreieich, Germany). Enzyme concentrations for astacin and meprin β were determined from the absorbance at 280 nm (∊_astacin_ = 42 800 *M* ^−1^ cm^−1^, ∊_meprin β_ = 106 520 *M* ^−1^ cm^−1^) and for ovastacin *via* the IC_50_ calculation of wild-type murine fetuin-B. Assays were performed in triplicate at 37°C in a 100 µl final volume, with 150 m*M* sodium chloride, 50 m*M* Tris–HCl pH 7.4, 0.01% Brij 35 as the buffer. Enzyme-activity measurements were started by the addition of 20–30 µ*M* Ac-RE(Edans)DRNleV­GDDP­YK(Dabcyl)-NH_2_ (Biosyntan GmbH, Berlin, Germany) for ovastacin and meprin β or 70–80 µ*M* Dansyl-PKRAPWV-OH (PANATecs GmbH, Heilbronn, Germany) for astacin dissolved in dimethyl sulfoxide (final concentration 0.4%). Initial velocities were recorded for at least 600 s (100 times for 100 ms at intervals of 15 s). Thereafter, 1.5 µl proteinase K (at 20 mg ml^−1^; Sigma–Aldrich, Taufkirchen, Germany) or 1 µl astacin (at 200 µ*M*) were added to reach complete substrate turnover, which was monitored and subsequently calculated using the formula *v* = [S] × *m*/Δ*F*, where [S] is the substrate concentration, *m* is the (*F*/*t*) slope of initial linear substrate turnover and Δ*F* is the maximal fluorescence intensity corresponding to complete turnover. Kinetic parameters of inhibition (*K*_i_) were determined using Morrison’s equation (Morrison, 1969 ▸).

### 2.5. Bioinformatics   

A computational homology model of human fetuin-A was calculated using the *automodel* routine of the *Modeller* v.9.20 suite (Fiser & Šali, 2003 ▸). The coordinates of mouse fetuin-B and a curated structure-based sequence alignment of the two proteins were used for these calculations, and the disulfides were fixed according to Kellermann *et al.* (1989 ▸). The resulting model was subjected to visual inspection with *Coot* to remove *cis*-peptide bonds and gross Ramachandran outliers, and was regularized with the *phenix.geometry_minimization* protocol of *PHENIX* with restraints to the starting model. The final model was assessed for the correct assignment of secondary-structure elements by comparison with an independent comparative model that was automatically generated using the multiple-template threading program *RaptorX* (http://raptorx.uchicago.edu/StructurePrediction/predict; Källberg *et al.*, 2012 ▸). The quality of the final model is reflected by the following statistics according to *PHENIX*: r.m.s.d.s from target values for bonds, angles, chirality and planarity of 0.002 Å, 0.57°, 0.04° and 0.01°, respectively, an all-atom clashscore of 9.7, 1.4% rotamer outliers and no *cis*-peptide bonds, and 12% and 88% of residues in allowed and favored Ramachandran regions, with 0% outliers. The coordinates are available from the last corresponding author upon request.

Sequences were aligned with *MUSCLE* (Edgar, 2004 ▸) as implemented in *SeaView* (Gouy *et al.*, 2010 ▸). The alignment was refined based on the structures of mouse fetuin-B overlaid with stefin-B (PDB entry 1stf; Stubbs *et al.*, 1990 ▸), cystatin-C (PDB entry 3gax; Kolodziejczyk *et al.*, 2010 ▸) and the aforementioned model of human fetuin-A using *UCSF Chimera* (Pettersen *et al.*, 2004 ▸). Structural figures were prepared with *UCSF Chimera*. Secondary-structure predictions were made with *JPred*4 (Drozdetskiy *et al.*, 2015 ▸). Structural superimpositions were performed with *SSM* (Krissinel & Henrick, 2004 ▸) within *Coot*. Protein interfaces were analyzed with *PISA* (Krissinel & Henrick, 2007 ▸; http://www.ebi.ac.uk/pdbe/pisa). The area of the complex interface was taken as half of the sum of the buried surface areas of the molecules. Sequence-similarity searches were performed with the *PSI-BLAST* protocol at NCBI (https://blast.ncbi.nlm.nih.gov/Blast.cgi) or the *BLAST* protocol at UniProt (https://www.uniprot.org/blast) using default parameters. Sequence identities were calculated using *SIM* with default parameters (https://web.expasy.org/sim/). The final coordinates of the crayfish astacin–mouse fetuin-B complex and free fetuin-B are available from the PDB at http://www.rcsb.org (PDB entries 6ht9 and 6hpv, respectively).

## 3. Results and discussion   

### 3.1. Crystallization of mouse fetuin-B   

Several groups have unsuccessfully tried to crystallize fetuins since their discovery (Pedersen, 1947 ▸) and ‘a number of obvious and potentially serious problems that may forestall attempts to crystallize fetuin’ were identified (Dziegielewska & Brown, 1995 ▸). Eventually, we obtained crystals of recombinant mouse fetuin-B from insect cells by incubation with an excess of the readily crystallizable MP astacin from crayfish (33% sequence identity with ovastacin; Gomis-Rüth, Trillo-Muyo *et al.*, 2012 ▸) as we failed to obtain sufficiently large amounts of ovastacin for structural studies. Astacin cleaved the inhibitor at S^296^–S^297^ within the CTR without affecting its inhibitory properties (data not shown). The resulting astacin–fetuin-B complex structure was solved by maximum-likelihood-scored molecular replacement, followed by density modification and noncrystallographic symmetry averaging, and was refined to 3.1 Å resolution (Table 1 ▸). Once the complex structure had been solved, we managed to obtain crystals of intact mouse fetuin-B from HEK293S cells, and the structure was independently solved by native sulfur phasing and refined to 2.3 Å resolution (Table 1 ▸). Examination of the crystal packing of the protein expressed in mammalian cells explains why it crystallized in its unbound form, whereas the material produced in insect cells did not: endoglycosidase H processing of the high-mannose N-glycans of the HEK293S-derived protein (a step performed during purification) left a single *N*-acetyl­glucosamine moiety attached to N^40^ that stacks against W^200^ of a symmetry-related molecule (Supplementary Fig. S1). This introduces a crucial crystal-packing contact that would most likely be hindered by the presence of additional sugar residues attached to the core *N*-acetylglucosamine, such as those normally found in proteins expressed in insect cells.

### 3.2. Structure of fetuin-B   

**Figure 1:**
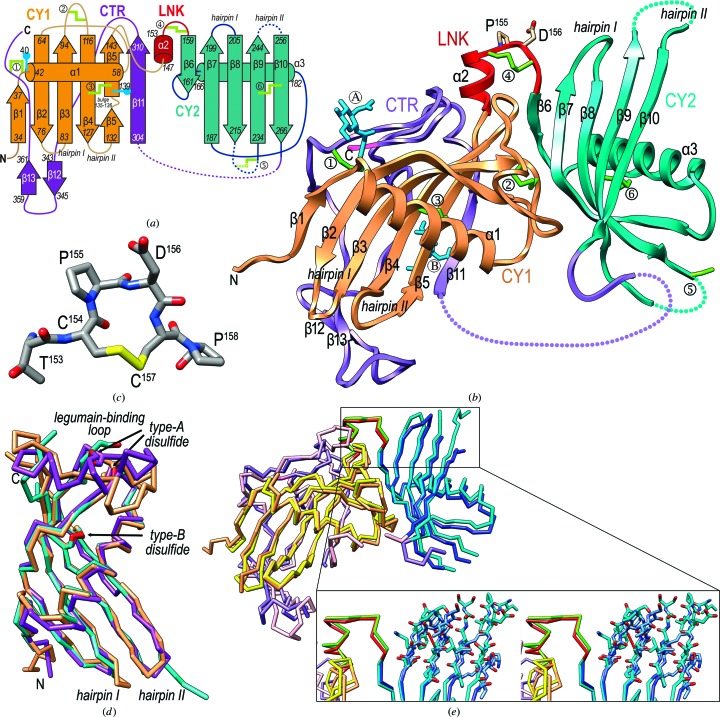
Structure of fetuin-B. (*a*) Topology scheme of mouse fetuin-B depicting strands as arrows (labeled β1–β13) and helices as rods (α1–α3), each with their delimiting residues. Domain CY1 is in orange, the linker (LNK) is in red, CY2 is in turquoise and the CTR is in purple. N-glycans are marked with blue lollipops and labeled. Disulfides are pinpointed as pale green lines and labeled: 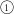, C^39^–C^374^; 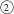, C^96^–C^107^; 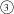, C^120^–C^140^; 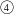, C^154^–C^157^; 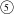, C^217^–C^224^; 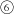, C^237^–C^263^. Missing segments are shown as dotted lines, and hairpins I and II of each cystatin domain are further labeled. (*b*) Ribbon-type plot of mouse fetuin-B, with each domain colored as in (*a*). N-glycans are labeled with an encircled ‘A’ (N^40^) and ‘B’ (N^139^); P^155^ and N^156^ from the CPDCP-trunk are labeled and their side chains are shown. The N- and the C-termini of the protein are labeled and residues from the purification tag are shown in pink. (*c*) The CPDCP-trunk featuring a rigid 14-atom ring (C atoms in gray). (*d*) Superposition of CY1 (orange), CY2 (cyan) and ovocystatin (purple; PDB entry 1cew; Bode *et al.*, 1988 ▸) as C^α^ plots. The respective type-A and type B-disulfides are shown in red and labeled, as are the legumain-binding loops in the three structures, hairpins I and II, and the N- and C-termini. (*e*) Superposition of the C^α^ traces of mouse fetuin-B: unbound (CY1 in yellow, LNK in green, CY2 in dark blue and CTR in pink) and in complex with astacin [domain colors as in (*b*)]. The inset shows a close-up showing the CPDCP-trunk, hairpin I and hairpin II in cross-eyed stereo. The observed differences may be authentic or may be the result of crystal contacts.

The structure of fetuin-B (residues R^29^–P^388^) in its astacin complex includes the domains and segments CY1 (R^29^–S^146^), linker (LNK; K^147^–P^158^) and CY2 (S^159^–E^267^, except for the loops T^218^–Q^227^ and L^246^–L^251^), and S^268^–V^272^ plus P^303^–P^388^ from the CTR (S^268^–P^388^). The N-terminal 118-residue CY1 domain exhibits the cystatin fold and consists of a twisted and curled five-stranded antiparallel β-sheet (strands β1–β5) with simple up-and-down connectivity. An α-helix (α1) is inserted between β1 and β2 through short linkers and nestles perpendicularly to the strands in the concave face of the sheet [Figs. 1 ▸(*a*) and 1 ▸(*b*)]. Strand pairs β2/β3 and β4/β5 are connected by short loops and are arranged as hairpins I and II, respectively. In contrast, β3 and β4 are connected by a long 21-residue loop (Lβ3β4), which was found in several conformations in ovocystatin (Engh *et al.*, 1993 ▸) and is involved in the inhibition of legumain (Dall *et al.*, 2015 ▸). This ‘legumain-binding loop’ is internally cross-linked by the disulfide C^96^–C^107^ (‘type-A disulfide’ according to Kellermann *et al.*, 1989 ▸). A second disulfide staples β4 and β5 together, roughly at half the strand length (C^120^–C^140^; type-B disulfide). A fifth cysteine (C^39^) in Lβ1α1 is bound to the CTR through a disulfide (see below), and two *N*-glycans are attached to N^40^ and N^139^, respectively. The C-terminal strand of CY1 (β5) contains a bulge and leads to the 12-residue LNK, which consists of a short α-helix (α2) and the segment C^154^PDCP^158^ arranged in a tight 1,4-turn of type I, hereafter referred to as the ‘CPDCP-trunk’ [Fig. 1 ▸(*c*)]. Owing to the prolines and the cysteines, which are disulfide-linked (C^154^–C^157^), this structure forms a rigid 14-atom ring that protrudes from the molecular surface [Fig. 1 ▸(*b*)]. In the absence of structural information, this disulfide had been previously termed type-C and included within the CY2 domain in fetuins and within the CY2 and CY3 domains in kininogen (Kellermann *et al.*, 1989 ▸; Lee, 2009 ▸).

The LNK is followed by the 109-residue CY2 domain, which likewise adopts the cystatin fold and is topologically equivalent to the CY1 domain. It interacts through the convex face of its β-sheet with Lα1β2 and the legumain-binding loop from the CY1 domain [Fig. 1 ▸(*b*)]. Interestingly, the latter loop encompasses the segment L^92^ETDCHVLSRKA^103^, which has been identified as a characteristic signature of fetuins (Olivier *et al.*, 2000 ▸) according to the PROSITE database (motif PDOC00966). This supports the hypothesis that the legumain-binding loop of the CY1 domain may play a similar adhesive role at the CY1/CY2 interface in other fetuins. CY1 and CY2 can be superimposed for 78 residues with an r.m.s.d. of 1.6 Å despite just 17% sequence identity, and the respective sheet strands (β6–β10 in CY2) and α-helices (α3 in CY2) coincide [Fig. 1 ▸(*d*)].

Akin to CY1 strands β4 and β5, a type-B disulfide (C^237^–C^263^) staples β9 and β10 together in the CY2 domain. The largest difference between the domains is found in the legumain-binding loop (Lβ8β9 in CY2), which spans 18 residues and is partially disordered in the CY2 domain, so that the corresponding type-A disulfide (C^217^–C^224^) is not resolved in the final Fourier map. Moreover, CY2 is not glycosylated, Lβ7β8 (hairpin I) is two residues shorter than in CY1 and Lβ9β10 (hairpin II) is partially disordered at its tip, as is Lβ8β9. Overall, the CY1 and CY2 domains are topologically equivalent to ovocystatin, except for the legumain-binding loop [Fig. 1 ▸(*d*); Bode *et al.*, 1988 ▸]: 83 and 97 aligned ovo­cystatin residues can be superposed with r.m.s.d. values of 2.1 and 2.0 Å onto CY2 and CY1 residues, respectively, despite low sequence-identity values (20% and 16%, respectively).

After the C-terminal strand of CY2 (β10), the polypeptide chain of fetuin-B enters the CTR, which is generally irregularly folded, disordered between P^273^ and A^302^, and cleaved at S^296^–S^297^. After P^303^, the chain runs parallel to strand β5 of CY1 as strand β11, thus providing a first anchor to CY1 for the downstream part of the domain. The remaining 78 residues of the CTR could be traced but are flexible: they lack regular secondary structure and run irregularly along the convex surface of the CY1 β-sheet. Only two short β-strands (β12 and β13), which coincide with secondary-structure predictions for the CTR, interact with each other in an antiparallel manner. Towards the C-terminus of the domain, C^374^ is disulfide-linked to C^39^, in this way providing a second anchor between the CTR and CY1. It is noteworthy that the tripeptide C^374^PG^376^ is conserved within fetuins, kininogens and histidine-rich glycoproteins (Olivier *et al.*, 2000 ▸). This suggests a similar link between CY1 and CTR in other fetuins, as experimentally proven for human fetuin-A and human kininogen (Kellermann *et al.*, 1989 ▸).

The structure of unbound intact mouse fetuin-B is defined for residues L^31^–P^388^ (except for the segments T^218^–H^228^, S^244^–V^255^, A^270^–T^295^ and E^316^–S^325^), and its superposition on the structure from the astacin complex reveals no significant conformational rearrangement [Fig. 1 ▸(*e*)]. The respective cores comprising CY1+LNK+CY2 are undefined for both the tip of hairpin II and Lβ8β9 of CY2, and they superpose with an r.m.s.d. of 1.7 Å. CY1 and CY2 are just rotated by ∼10° with respect to each other when going from the unbound to the complexed structure. The only functionally relevant differences in the cores are found at the tips of hairpins I and II of CY2 [inset in Fig. 1 ▸(*e*)], which however are very likely to result from crystal contacts in the unbound structure. Hairpin I is in a noncompetent conformation for binding, and becomes folded out towards the CPDCP-trunk upon complex formation. In addition, despite being disordered in both structures, hairpin II shows some extra ordered residues in the complex structure, possibly owing to fixing interactions with the target MP (see below). Although the CTR of unbound fetuin-B is intact and that of the complexed form is cleaved (see above), they show similar overall chain traces (except for S^348^–K^357^, D^314^–E^328^ and the C-terminal tail after N^381^), as revealed by an r.m.s.d. of 1.3 Å upon superposition.

### 3.3. Structure-based mechanism of the inhibition of astacin by fetuin-B   

Astacin is a 202-residue MP (A_1_–H_202_). It consists of an upper subdomain (USD; A_1_–G_99_) and a lower subdomain (LSD; F_100_–H_202_) separated by an extended, deep, narrow active-site cleft (Bode *et al.*, 1992 ▸; Gomis-Rüth *et al.*, 1993 ▸; Guevara *et al.*, 2010 ▸). The USD contains a twisted five-stranded β-sheet, the fourth strand of which shapes the upper rim of the cleft and runs antiparallel to the other strands and to substrates bound in the cleft. The USD further contains an active-site helix. This helix includes two of the catalytic zinc ligands and the general base/acid for catalysis (H_92_, H_96_ and E_93_, respectively) embedded in a long zinc-binding consensus sequence (H_92_E*XX*H*XX*G*XX*H_102_), which is a hallmark of the astacins (Gomis-Rüth, Trillo-Muyo *et al.*, 2012 ▸) and other MPs of the metzincin clan (Bode *et al.*, 1993 ▸; Gomis-Rüth, 2009 ▸; Cerdà-Costa & Gomis-Rüth, 2014 ▸). After G_99_, the chain enters the LSD, which contains little repetitive secondary structure, most notably a kinked C-terminal helix at the end of the chain. Two further zinc ligands come from the LSD (H_102_ and Y_149_), as well as another structural hallmark of astacins and other metzincins, the Met-turn (Tallant *et al.*, 2010 ▸), which contains a conserved methionine (M_147_). The C-terminus of the protein resides on the molecular surface and two disulfides (C_42_–C_198_ and C_64_–C_84_) contribute to the overall stability and rigidity of the molecule.

**Figure 2:**
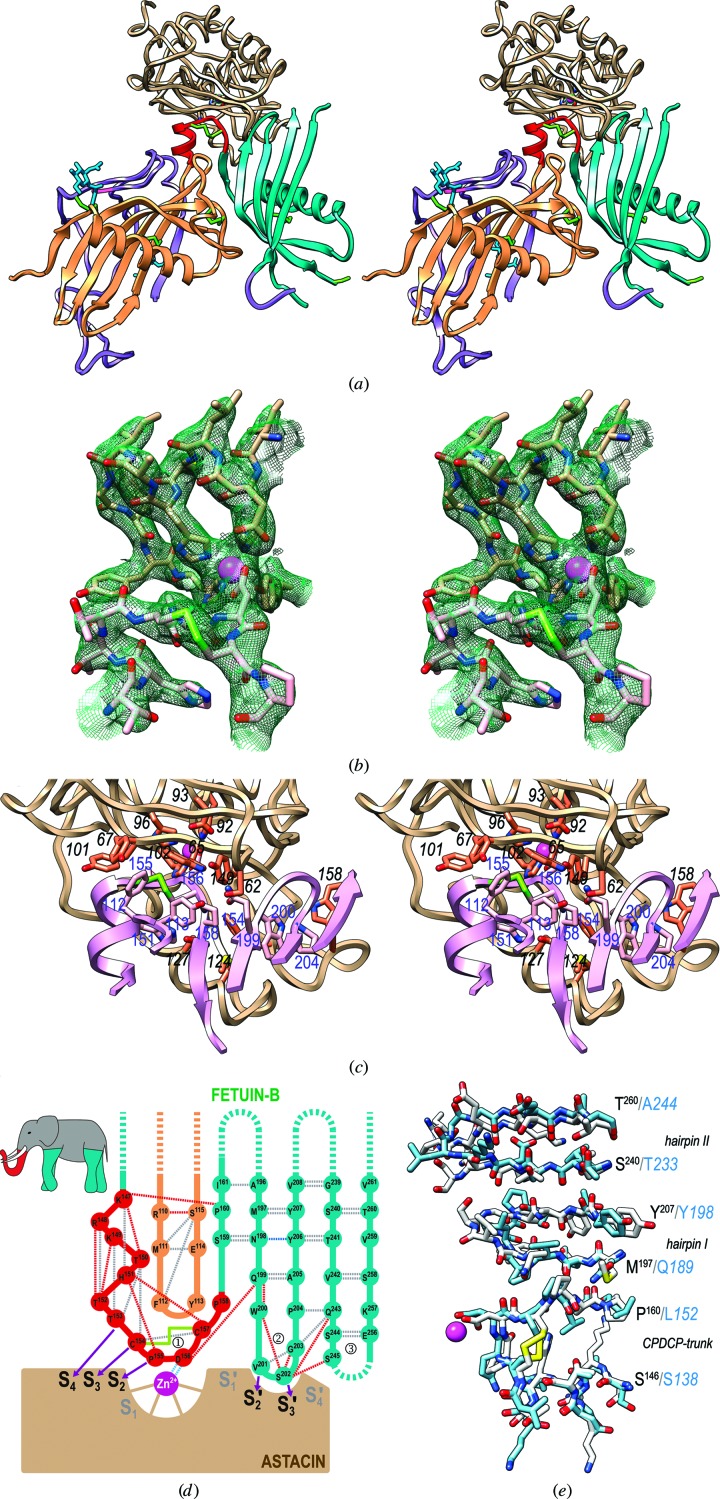
The astacin–fetuin-B complex. (*a*) Cross-eyed stereo cartoon displaying fetuin-B [colors as in Fig. 1 ▸(*b*)] inserted into the active-site cleft of astacin (tan ribbon). The MP is shown after a horizontal ∼90° rotation downwards from the traditional standard orientation of MPs (Gomis-Rüth, Botelho *et al.*, 2012 ▸), so the cleft runs from left (nonprimed subsites) to right (primed subsites). The catalytic zinc is shown as a magenta sphere and its astacin ligands as stick models, as are P^155^ and D^156^ from fetuin-B. (*b*) Detail of the final (2*mF*_obs_ − *DF*_calc_)-type Fourier map at 3.1 Å resolution contoured at 1σ above the threshold, depicting the zinc-binding site of astacin (carbons in tan, zinc ion as a magenta sphere) and the CPDCP-trunk of fetuin B (carbons in pink). (*c*) Close-up view of (*a*) after a horizontal 35° rotation, displaying selected residues participating in the interaction between fetuin-B (C^α^ ribbon in plum; from left to right, segments S^146^–D^162^, M^111^–E^114^, N^197^–Y^206^ and T^241^–S^245^; carbons in pink and residue numbers according to UniProt Q9QXC1 in blue) and astacin (C^α^ ribbon in tan, carbons in orange, catalytic zinc as a magenta sphere and residue numbers in black italics according to UniProt P07584). The C^154^–C^157^ disulfide from the CPDCP-trunk of fetuin-B is shown with green sulfurs. (*d*) Scheme of the proposed ‘raised elephant-trunk model’; the view mirrors that in (*a*) and (*b*). The CPDCP-trunk (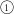) would be the raised trunk (in red) and hairpin I (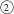) and II (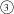) the two front and back limbs (in turquoise), respectively (see top-left inset). The trunk contacts the catalytic zinc ion (magenta sphere) of astacin (in brown) by residues nestling into cleft subsites **S_4_**, **S_3_**, **S_2_**, **S_2_′** and **S_3_′**, as shown by purple arrows. Dashed lines represent distantly connected segments or disordered loops. Hydrogen bonds between main-chain atoms, between side-chain and main-chain atoms, and between side-chain atoms are shown as gray, red and blue dashed lines, respectively. The electrostatic interaction between the catalytic zinc and D^156^ is in light blue. The disulfide connecting the CPDCP-trunk is shown as a green solid line. (*e*) Superposition of the experimental structure of mouse fetuin-B (white carbons and black labels) and the comparative model of human fetuin-A (cyan carbons and blue labels with regular residue numbers in italics) depicting the regions of the CPDCP warhead and the tips of hairpins I and II of each structure. The view results from (*c*) after a 90° rotation in the plane. The catalytic zinc ion of astacin is further shown as a magenta sphere for reference.

Mouse fetuin-B potently inhibits its physiological target ovastacin, human meprin β and archetypal crayfish astacin *in vitro* (see Section 3.4 and Karmilin *et al.*, 2019 ▸). To account for this inhibition, the LNK and CY2 form a bipartite wedge, which slots into the active-site cleft of the target [Figs. 2 ▸(*a*), 2 ▸(*b*) and 2 ▸(*c*)]. In contrast, CY1 makes only a minor contribution to binding and the CTR does not participate at all. Upon inhibitor binding, the LSD of astacin undergoes a slight clamshell motion of maximally ∼2.5 Å (at the C^α^ atom of Q_118_), which closes the active-site cleft as in the complex with an inhibitor mimicking a reaction intermediate (PDB entry 1qji; Grams *et al.*, 1996 ▸). The astacin–fetuin-B complex interface buries an area of 1059 Å^2^ and yields a theoretical gain of solvation energy on complex formation of −10.8 kcal mol^−1^. The interface involves 33 residues of fetuin-B and 38 of astacin, which collectively establish ten hydrogen bonds, two ionic interactions and 20 hydrophobic interactions (see Table 2 ▸). Astacin segments participating in the interaction include S_62_–V_68_ from the upper-rim strand and a preceding bulge above the primed side of the cleft, Q_76_ from the fifth strand, G_83_–C_84_ from the loop connecting the fifth sheet strand to the active-site helix, plus the disulfide C_64_–C_84_, the zinc-binding site and Y_101_, D_121_–D_129_, F_154_–W_158_ and Y_177_ from the LSD [Fig. 2 ▸(*c*)]. Fetuin-B segments involved include M^111^–Y^113^ from the legumain-binding loop of CY1, K^147^–S^159^ from the LNK and CY2 strand β6, N^198^–Y^206^ from CY2 hairpin I and, to a lesser extent, S^245^ plus Q^253^–V^255^ from CY2 hairpin II, the tip of which is disordered [see above and Figs. 2 ▸(*a*) and 2 ▸(*c*)].

The main interaction is performed by the rigid CPDCP-trunk of the LNK, which blocks the nonprimed side of the cleft and binds the catalytic zinc through the side chain of D^156^ in a bidentate manner, thus occupying the position of a scissile carbonyl during catalysis. The D^156^ carboxylate is also at binding distance from the zinc-binding atom Y_149_ O^η^ and, notably, the carboxylate of the general base/acid E_93_, so that either carboxylate could be protonated. P^155^ nestles into cleft subsite **S_2_** [active-site cleft subsites are shown in bold; for nomenclature, see Schechter & Berger (1967 ▸) and Gomis-Rüth, Botelho *et al.* (2012 ▸)], C^154^ into **S_3_** and T^153^ into **S_4_**. The cysteine sulfurs of the trunk make hydrophobic interactions with the ring of W_65_. In addition, residues M^111^–Y^113^ from the legumain-binding loop of CY1 play a scaffolding role for the CPDCP-trunk and further establish mixed hydrophobic and hydrophilic interactions with S_62_, W_65_, Y_67_ and Q_76_ of astacin [see Table 2 ▸ and Figs. 2 ▸(*c*) and 2 ▸(*d*)].

Next in importance to complex formation is CY2 hairpin I, which blocks the primed side of the cleft. The Q^199^ side chain both positions the CPDCP-trunk through a hydrogen bond to D^156^ O and binds the main chain of the astacin bulge at S_62_ O. The downstream residue W^200^ makes a hydrophobic inter­action through one face of its side chain with F_154_ and M_124_; through the other it is kept in place by the side chain of P^204^ upstream in the hairpin. The V^201^ side chain occupies **S_2_′** and interacts hydrophobically with the sulfurs of the disulfide C_64_–C_84_, while S^202^ enters **S_3_′** [Figs. 2 ▸(*c*) and 2 ▸(*d*)]. Owing to its missing side chain, the downstream residue G^203^ is essential to prevent a clash with W_158_ of astacin. Moreover, Y^206^ establishes a hydrogen bond to D_121_. Overall, these interactions are key for complex formation and comprise a motif for the tip of CY2 hairpin I (Q^199^WVSGP^204^), which is conserved as QWV*X*GP among mammalian orthologs (see below, Table 3 ▸ and Supplementary Figs. S2 and S3). Finally, weak interactions are provided by the defined residues of CY2 hairpin II. In particular, S^245^ and Q^253^–V^255^ are close to Y_177_ and Q_157_–V_160_ of astacin.

Overall, the CPDCP-trunk and CY2 hairpin I both bind in the direction of a substrate and cover the nonprimed and primed sides of the active-site cleft from **S_4_** to **S_3_′**. This matches the preference of the enzyme for elongated substrates (Grams *et al.*, 1996 ▸) and explains the high inhibitory potency of fetuin-B. However, subsites **S_1_** and **S_1_′** are spared by the inhibitor, which is noteworthy as **S_1_′** is the main specificity pocket of astacins and most MPs (Gomis-Rüth, Botelho *et al.*, 2012 ▸; Gomis-Rüth, Trillo-Muyo *et al.*, 2012 ▸). This enables fetuin-B to potently inhibit astacin, meprin β and ovastacin (see Section 3.4), despite their differences in substrate specificity (Gomis-Rüth, Trillo-Muyo *et al.*, 2012 ▸), and strongly supports astacin–fetuin-B as a valid model for other complexes with members of the astacin family of MPs. The free **S_1_′** pocket may also explain why the zinc ligand Y_149_, which undergoes a ‘tyrosine-switch’ motion upon substrate or inhibitor binding (Grams *et al.*, 1996 ▸; Gomis-Rüth, Trillo-Muyo *et al.*, 2012 ▸), is found in the conformation present in unbound astacin structures and the proenzyme, which likewise evince empty **S_1_′** pockets (Gomis-Rüth *et al.*, 1993 ▸; Guevara *et al.*, 2010 ▸). Thus, the bipartite blockage of the cleft prevents cleavage of the inhibitor, as occurs for example in standard-mechanism inhibitors of MPs, which typically run entirely along the cleft (Arolas *et al.*, 2011 ▸). In addition, this mode of inhibition also explains why fetuin-B does not inhibit BMP-1 and its tolloid(-like) relatives (Karmilin *et al.*, 2019 ▸): these proteins have astacin residue W_65_, which is located within the upper-rim strand above the cleft, replaced by the tripeptide CCG, with the two vicinal cysteines connected by a *cis*-peptide bond and a disulfide bond. This creates a highly strained bulge above the zinc site (see PDB entry 3edh; Mac Sweeney *et al.*, 2008 ▸), which prevents the fetuin-B CPDCP-trunk from entering the active site. Moreover, the mechanism also explains why CY1 is not inhibitory: despite its overall structural similarity (see Section 3.2), CY1 lacks an upstream CPDCP-trunk and its hairpin I is two residues longer at its tip [Fig. 1 ▸(*d*)]. This impairs the functionality of the QWV*X*GP motif (H^76^YQEDMGS^83^ in CY1) and would cause collision with the primed side of the astacin cleft. Finally, CY1 hairpin II is seven residues shorter than in CY2, so that even the minor contribution to inhibition by this structural element is missing. To sum up, the CPDCP-trunk and CY2 hairpin I are the major determinants of inhibition, while CY1 and CTR merely play a scaffolding role.

As to structurally similar inhibitors, human latexin likewise consists of tandem cystatin-like domains connected by a helical linker (PDB entry 2bo9; Pallarès *et al.*, 2005 ▸). Latexin is also very specific to a particular MP family, here the A/B-type funnelins, which are also known as M14 metallocarboxy­peptidases (Gomis-Rüth, 2008 ▸). Like fetuin-B, it inhibits its targets through the C-terminal cystatin domain. However, in latexin the two cystatin domains are packed against each other through the helices, which are surrounded by the curved β-sheets arranged in a compact open barrel. In addition, latexin inhibition is not exerted by segments that are topologically reminiscent of the CPDCP-trunk plus hairpins I and II, but rather through loops from the opposite edge of the sheet, equivalent to Lβ6α3, Lα3β7 and Lβ8β9 of fetuin-B CY2. In summary, despite the similarity in the building blocks, the overall structure and the working mechanism of fetuin-B are novel for MPs.

### 3.4. Experimental validation of the structure-derived mechanism   

**Figure 3:**
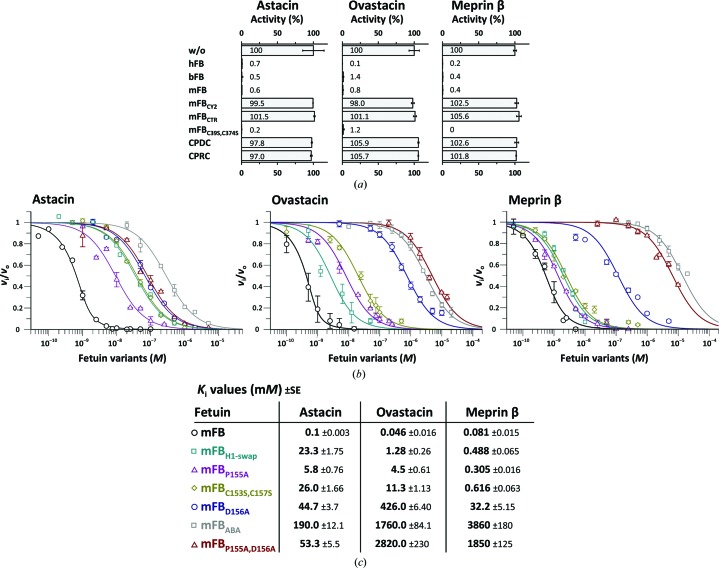
*In vitro* validation of the inhibitory mechanism. (*a*) Activity (%) of astacin (at 1 n*M* concentration), meprin β (at 1 n*M* concentration) and ovastacin (at 5.6 n*M*) in the presence of fetuin-B variants at 300 n*M* (CPDC and CPRC) or 12.5 n*M* (other variants). Full astacin, meprin β and ovastacin activities (100%) correspond to substrate-turnover rates of 35.4 ± 5.7, 7.7 ± 0.2 and 10.7 ± 0.8 n*M* s^−1^, respectively; w/o, without fetuin; hFB, human fetuin-B; bFB, bovine fetuin-B; mFB, murine fetuin-B; mFB_CY2_, CY2 domain of mFB; mFB_CTR_, CTR domain of mFB; mFB_C39S,C374S_, double point mutant of murine fetuin-B; CPDC and CPRC, cyclic peptides comprising the respective sequences. Experiments were performed in triplicate; error bars indicate standard deviations. (*b*) Plot of fractional velocity (logarithmic scale) with different fetuin-B variants: mFB, murine fetuin-B; mFB_ABA_, variant with CY1 and CTR from mouse fetuin-A and CY2 from mFB; mFB_H1-swap_, mutant replacing CY2 hairpin I with CY1 hairpin I; mFB_P155A_, mFB_D156A_, mFB_P155A,D156A_ and mFB_C154S,C157S_ are point mutants of murine fetuin-B. Enzyme and substrate concentrations were 1.0 n*M* and 195 µ*M* for astacin, 0.5 n*M* and 26 µ*M* for meprin β and 0.7 n*M* and 26 µ*M* for ovastacin, respectively. Error bars indicate standard deviations. (*c*) *K*_i_ values determined from (*b*) using Morrison’s equation with the respective standard errors (SE).

To verify the abovementioned mechanism, we tested the inhibition of several constructs and mutants of human, mouse and bovine fetuin-B against crayfish astacin, human meprin β and mouse ovastacin [Fig. 3 ▸(*a*)], which add to previous studies (Karmilin *et al.*, 2019 ▸). We also determined the constants of inhibition (*K*_i_) of selected constructs and mutants of mouse fetuin-B to provide quantitative data [Figs. 3 ▸(*b*) and 3 ▸(*c*)].

The three full-length wild-type orthologs completely inhibited all enzymes [Fig. 3 ▸(*a*)], in accordance with previous studies (Karmilin *et al.*, 2019 ▸). Wild-type mouse fetuin-B inhibited astacin, meprin β and ovastacin with similar *K*_i_ values of 100, 81 and 46 p*M*, respectively [Fig. 3 ▸(*c*)]. In contrast, the *K*_i_ values of a chimera of mouse fetuin-B, in which CY1 and CTR were replaced by the homonymous domains of non-inhibitory mouse fetuin-A (mutant mFB_ABA_), was decreased by more than three (astacin) and four (meprin β and ovastacin) orders of magnitude, respectively [Figs. 3 ▸(*b*) and 3 ▸(*c*)]. The mutants mFB_BAA_ and mFB_BAA_ could not be produced, which underpins the requirement for a complementary CY1–CTR interface for the proper folding of fetuin-B.

A cyclic peptide including a sequence CPDC, which mimics the isolated CPDCP-trunk between CY1 and CY2, did not inhibit either enzyme and was indistinguishable from a variant with an arginine replacing the aspartate [Fig. 3 ▸(*a*)]. Taken together with the lack of inhibition of isolated domain CY2 [Fig. 3 ▸(*a*)], this indicates that these modules are only functional within the context of a multi-domain fetuin. Domain CTR alone did not show inhibition either [Fig. 3 ▸(*a*)], which is consistent with recombinant carp fetuin truncated after CY2 inhibiting meprin α/β and carp nephrosin with similar potency as the functional full-length protein purified from fish blood (Hedrich *et al.*, 2010 ▸). Together, these data point to a negligible role of the CTR in inhibition. Consistently, no reduction of activity was observed when the cysteines of disulfide C^39^–C^374^ were replaced by serines [Fig. 3 ▸(*a*)], which indicates that the minimal functional structure does not require the overarching clamp between CY1 and CTR.

Point mutations affecting the trunk within full-length mouse fetuin-B all had negative effects on inhibition. The P^155^A mutant caused a decrease in the *K*_i_ values of roughly one order of magnitude for astacin and meprin β and two orders of magnitude for ovastacin. For the D^156^A mutant, we measured an approximate decrease of two orders of magnitude for astacin and meprin β and four orders of magnitude for ovastacin. The corresponding double mutant caused an even greater loss towards ovastacin and meprin β (four orders of magnitude) and astacin (two orders of magnitude) [Figs. 3 ▸(*b*) and 3 ▸(*c*)]. Thus, similar to the mFB_ABA_ mutant, the double mutant is merely a micromolar inhibitor [Fig. 3 ▸(*c*)] that is insufficient to block ovastacin under physiological conditions. Likewise, the C^154^S+C^157^S mutant led to a substantial decrease in *K*_i_. Finally, grafting the sequence of CY1 hairpin I onto CY2 (H1-swap mutant; QEDMGP instead of QWVSGP) also led to substantially higher *K*_i_ values [Figs. 3 ▸(*b*) and 3 ▸(*c*)]. This indicates that the conserved Q*X*V*X*GP sequence motif of H1 is required for efficient inhibition of astacins.

Overall, the fact that the mutants and variants have comparable effects on astacin, meprin β and ovastacin supports the crayfish enzyme as a valid structural model for the mammalian orthologs. The results confirm that the major functional interactions between fetuin-B and astacin/ovastacin are made by the CPDCP-trunk and hairpin I of domain CY2 and that any modification leads to a decrease in inhibitory power. Moreover, an overall scaffold encompassing CY1+LNK+CY2 is indispensable to exert full inhibitory potency.

### 3.5. Comparison with the inhibition mechanism of cysteine peptidases by cystatins   

The inhibitory mechanism of cysteine peptidases by monomeric cystatins has been dubbed the ‘elephant-trunk model’ (Turk & Bode, 1991 ▸) and is mainly exerted by the N-terminal segment of the inhibitor (the ‘trunk’). This segment precedes the first strand of the β-sheet and occupies the **S_1_** and upstream nonprimed subsites of the cleft (Stubbs *et al.*, 1990 ▸; Turk & Bode, 1991 ▸). On the primed side, hairpin I contains the conserved sequence Q*X*V*X*G, which functions similarly to hairpin I of fetuin-B with motif QWV*X*GP (see above). Finally, hairpin II of cystatin provides a scaffold for hairpin I and performs ancillary interactions with the enzyme through the conserved PW motif. Overall, the inhibitory mechanism of MPs by fetuin-B, in which the extended N-terminal trunk is replaced by a compact CPDCP-trunk that resembles a ‘raising trunk’ [inset in Fig. 2 ▸(*d*)], contains elements of similarity to the hydrophobic tripartite wedge of cystatins tackling cysteine peptidases. This suggests that gene duplication and molecular evolution of cysteine-peptidase targeting cystatins eventually led to inhibition of peptidases of a different chemical class. We thus hereby propose the term ‘raised-elephant-trunk mechanism’ to describe the mode of inhibition of astacins by fetuin-B [Fig. 2 ▸(*d*)].

### 3.6. Implications for inhibitory fetuins   

The alignment of representative sequences of (potential) mammalian fetuin-B orthologs revealed that they share the same overall length (374–388 residues) and show high sequence identity to the human form, which ranges from 98% (gorilla) to 47% (opossum) (see Table 3 ▸). The orthologs contain the sequence of the CPDCP-trunk and the length and sequence of the QWV*X*GP motif (Table 3 ▸, Supplementary Figs. S2 and S3), as well as a similar CTR. This suggests that mammalian fetuin-B orthologs probably exhibit equivalent structures and inhibit astacins *via* the proposed raised-elephant-trunk mechanism, as has been confirmed to date for the bovine, human and murine forms [Fig. 3 ▸(*a*)].

Outside mammals, carp fetuin efficiently inhibited nephrosin (Tsai *et al.*, 2004 ▸) and human meprin α and β, but not astacin (Hedrich *et al.*, 2010 ▸). This fetuin occurs in two isoforms, which have a CTR that is either shorter or longer than that in mouse fetuin-B but is in both cases dispensable for inhibition (Hedrich *et al.*, 2010 ▸). Although somewhat more distant in evolution than the mammalian orthologs (24% sequence identity between the first 306 residues of mouse fetuin-B and the entire short variant), carp fetuin also contains the CPDCP-trunk to bind the catalytic zinc, but hairpin I has one extra residue in the sequence: QWMFSGQ. This would entail steric clashes with the primed side of the cleft of astacin, but not of meprin β (PDB entry 1gwn; Arolas *et al.*, 2012 ▸), the cleft of which is deeper. This explains the distinct inhibitory behavior towards these enzymes.

Other vertebrates (birds, reptiles, amphibians, bony fishes and cartilaginous fishes) contain potential orthologs with both the CPDCP-trunk and a putative hairpin I with a QWV*X*GP-derived sequence (Table 3 ▸). They should thus indiscriminately inhibit meprins, astacin and ovastacin. However, the CTRs strongly deviate from mammalian sequences and range from short tails to hundreds of residues. In particular, the Chinese softshell turtle sequence only spans 264 residues and is apparently a natural variant containing just CY1+LNK+CY2. Moreover, no sequences were found within cyclostomes, cephalochordates, urochordates, echinoderms or hemichordates within deuterostomes. Thus, fetuin-B sequences are absent from jawless fish and are restricted to jawed vertebrates, for which the raised-elephant-trunk mechanism might be valid.

### 3.7. Why fetuin-A is not an astacin inhibitor   

To investigate the lack of inhibitory activity of fetuin-A (Karmilin *et al.*, 2019 ▸), we calculated a homology model for the human form based on the coordinates of mouse fetuin-B (28% sequence identity) and the experimentally determined disulfide pattern (Kellermann *et al.*, 1989 ▸). According to this model, the overall structure would be very similar to that of fetuin-B and likewise would encompass CY1 (G*22*–S*138*; human fetuin A residue numbers are shown in italics; see UniProt P02765), LNK (A*139*–P*150*), CY2 (L*151*–Q*251*) and CTR (T*252*–V*367*) domains. It would also include a potential CPDCP-trunk (C*146*QDCP*150*), which would be solvent-exposed, as well as the upstream major physiological trypsin cleavage site of human fetuin-A (K*143*–K*144*; Kellermann *et al.*, 1989 ▸). Moreover, the segments equivalent to Y^77^–Q^78^ and H^151^TT^153^ of fetuin-B are sensitive to proteolysis in some fetuin-A orthologs (Olivier *et al.*, 2000 ▸, Kellermann *et al.*, 1989 ▸). These segments map to CY1 hairpin I and helix α3, respectively, which are likewise surface-located.

The second position of the CPDCP-trunk is occupied by a glutamine in human fetuin-A (Q*147*), which could potentially interfere with active-site residues of the MP and thus impair inhibition. In contrast to the fetuin-B orthologs, the trunk motif shows variability across mammalian fetuin-A orthologs: CPRCP in mouse, CPQCP in cat, CPNCP in pig *etc*. In the bovine and rabbit orthologs the sequence even corresponds to that of fetuin-B. Collectively, these findings support the above results *in vitro* that the ‘warhead’ is a necessary but not sufficient element of inhibition. Indeed, the main reason for the lack of inhibition of human fetuin-A is that CY2 hairpin I is one residue shorter, shows a disparate sequence (V*191*PL­PP*195* instead of QWV*X*GP) and probably shows a distorted geometry owing to the two extra prolines [Fig. 2 ▸(*e*)]. In addition, the CY2 hairpin II would be nine residues shorter (K*237*–G*240* instead of S^244^–E^256^). Taken together, these differences in crucial and ancillary structural elements of the raised-elephant-trunk model would explain the lack of inhibitory competence of fetuin-A.

## 4. Conclusion   

Discovered over seven decades ago in mammalian serum, the fetuins are a widespread family of vertebrate proteins. Fetuin-B is the only endogenous, specific, potent inhibitor of non-BMP-1-like astacin MPs through a conserved aspartate within a CPDCP-trunk that blocks the active-site cleft. This is reminiscent of the aspartate-switch mechanism that is responsible for latency in astacin zymogens, in which an aspartate likewise blocks the catalytic zinc (Guevara *et al.*, 2010 ▸; Arolas *et al.*, 2012 ▸).

CY1, LNK and CY2 are required for the inhibitory function of fetuin-B, but not the CTR, which is flexible and depleted of regular secondary structure, *i.e.* it can potentially adopt several conformations. By lining the exposed surface of CY1 and being covalently linked to it, the CTR provides fetuins with an adaptable surface to potentially bind many proteins and exert disparate signaling functions (Dunker *et al.*, 2000 ▸), which may be independent of inhibition of proteolysis.

Finally, we found sequences of proven and potential fetuin-B orthologs encompassing the CPDCP and QWV*X*GP motifs throughout jawed vertebrates down the tree of life as far as cartilaginous fish. In turn, astacins are present across animals, including lower vertebrates and invertebrates such as nematodes, mostly as several paralogs per organism (Gomis-Rüth, Trillo-Muyo *et al.*, 2012 ▸). This phylogenetic coexistence suggests that the raised-elephant-trunk mechanism described here may represent a general mechanism of endogenous regulation of astacin MPs through dedicated and potent protein inhibitors.

## Supplementary Material

PDB reference: mouse fetuin-B, 6hpv

PDB reference: complex with astacin, 6ht9

Supplementary figures.. DOI: 10.1107/S2052252519001568/jt5031sup1.pdf

## Figures and Tables

**Table 1:** Crystallographic data Values in parentheses are for the outermost resolution shell.

Data set	Astacin–fetuin-B	Fetuin-B
Space group	*P*2_1_2_1_2_1_	*P*4_1_2_1_2
*a*, *b*, *c* (Å)	83.4, 85.8, 168.7	67.7, 67.7, 197.8
Wavelength (Å)	0.9792	1.7712
No. of measurements	264256	384912
No. of unique reflections	22647	19993
Resolution range (Å)	84.4–3.10 (3.28–3.10)	47.2–2.30 (2.38–2.30)
Completeness (%)	100.0 (100.0)	93.6 (63.0)
*R*_merge_	0.153 (1.499)	0.109 (1.626)
*R*_meas_	0.160 (1.567)	0.112 (1.782)
CC_1/2_	0.998 (0.771)	0.999 (0.521)
Average intensity	14.1 (2.0)	20.3 (1.4)
*B* factor (Wilson) (Å^2^)	78.8	54.2
Avererage multiplicity	11.7 (11.6)	19.3 (6.0)
No. of reflections used in refinement	21981	19989
No. of reflections in test set	643	1000
*R* factor/free *R* factor	0.216/0.270	0.224/0.254
Correlation coefficient, *F*_obs_ − *F*_calc_	0.931	0.931
Correlation coefficient, test set	0.882	0.939
No. of protein residues	1127	299
No. of atoms	7899	2344
No. of solvent molecules	46	85
No. of covalent ligands	5 NAG†	2 NAG†
No. of noncovalent ligands	2 Zn^2+^, 3 glycerol	1 acetate
R.m.s.d. from target values		
Bonds (Å)	0.010	0.002
Angles (°)	1.14	0.59
Average *B* factors (Å^2^)		
Overall	101.2	67.1
Molecule *A*	79.2	67.7
Molecule *B*	109.0	—
Molecule *C*	89.6	—
Molecule *D*	117.1	—
All-atom contacts and geometry analysis		
Residues in favored regions	908 [91.6%]	280 [96.7%]
Outliers	7	0
All residues	991	290
Outlying rotamers	56 [6.4%]	5 [1.8%]
Outlying bonds	0	0
Outlying angles	0	0
Outlying chirality	0	0
Outlying planarity	0	0
All-atom clashscore	4.8	3.2

† *N*-acetyl-D-glucosamine.

**Table 2:** Interactions at the astacin–fetuin-B interface The first residue/atom belongs to fetuin-B and the second to astacin. The overall resolution of the structure is 3.1 Å, the estimated overall average coordinate error is 0.42 Å based on the Luzzati plot, and the dispersion precision indicator based on the free *R* factor is 0.49 Å. Thus, the distances are approximate.

Hydrogen bonds
Y^113^ O^η^–S_62_ O^γ^, 3.0 Å
H^151^ N^δ1^–N_127_ O^δ1^, 3.1 Å
T^152^ O–Y_101_ O^η^, 2.9 Å
T^153^ O–V_68_ N, 2.7 Å
D^156^ O^δ1^–Y_149_ O^η^, 2.7 Å
D^156^ O^δ2^–E_93_ O^∊2^, 2.9 Å
Q^199^ N^∊2^–S_62_ O, 3.1 Å
S^202^ O–Y_177_ O^η^, 3.9 Å
S^202^ O–W_158_ N^∊1^, 3.6 Å
Y^206^ O^η^–D_121_ O^δ1^, 3.3 Å
Ionic interactions
D^156^ O^δ1^–Zn_999_, 2.3 Å
D^156^ O^δ2^–Zn_999_, 2.3 Å
Hydrophobic interactions (<4 Å)
F^112^–W_65_
F^112^–Y_67_
C^154^–W_65_
P^155^–V_68_
P^155^–H_96_
P^155^–H_102_
D^156^–W_65_
C^157^–W_65_
P^158^–W_65_
N^198^–M_124_
Q^199^–G_63_
W^200^–M_124_
W^200^–F_154_
V^201^–G_63_
V^201^–C_64_
V^201^–G_83_
V^201^–C_84_
C^154^–W_65_
S^202^–Y_177_
S^245^–W_158_

**Table 3:** Potential fetuin-B orthologs in vertebrates All sequences contain the intact CPDCP-trunk motif. UP, UniProt; GB, GenBank. Sequence identity with human fetuin-B was determined only for mammalian sequences, as they share the same overall protein length and architecture. Other vertebrate sequences share domains CY1 and CY2 but have highly variable CTRs.

Organism	Database code	Sequence identity† (%)	Hairpin I sequence
Mammals			
Human (*Homo sapiens*)	UP Q9UGM5	100	QWVVGP
Western lowland gorilla (*Gorilla gorilla*)	UP G3QMB1	98	QWVVGP
Chimpanzee (*Pan troglodytes*)	UP A0A2J8M5X2	97	QWVVGP
Sumatran orangutan (*Pongo abelii*)	UP A0A2J8WHT1	97	QWVVGP
Fruit bat (*Pteropus vampyrus*)	GB XP_011380247	75	QWVFGP
African elephant (*Loxodonta africana*)	UP G3SVW6	70	QWVVGP
Dog (*Canis lupus familiaris*)	UP E2R9B6	69	QWVYGP
Sperm whale (*Physeter catodon*)	GB XP_007116341	69	QWVFGP
Naked mole rat (*Heterocephalus glaber*)	UP G5BT88	67	QWVVGP
European hedgehog (*Erinaceus europaeus*)	GB XP_007519827	66	QWVFGP
Bovine (*Bos taurus*)	UP Q58D62	66	QWVFGP
Horse (*Equus caballus*)	UP F6RRV1	63	QWVVGP
Mouse (*Mus musculus*)	UP Q9QXC1	63	QWVSGP
Rat (*Rattus norvegicus*)	UP Q9QX79	61	QWVVGP
Pig (*Sus scrofa*)	UP F1SFI6	60	QWVFGP
Koala (*Phascolarctus cinereus*)	GB XP_020844617	50	QWVFGP
Opossum (*Monodelphis domestica*)	GB XP_001373317	47	QWVVGP
Birds			
Mallard (*Anas platyrhynchos*)	UP U3IFE7		QMVIGP
Zebra finch (*Taeniopygia guttata*)	UP H0ZJK5		QWVIGP
Rock dove (*Columba livia*)	UP F1NHT5		QWVIGP
Collared flycatcher (*Ficedula albicollis*)	UP U3K0K6		QWVVGP
Reptiles			
American chameleon (*Anolis carolinensis*)	UP H9GD35		QWVVGP
Bearded dragon (*Pogona vitticeps*)	GB XP_020666227		QWVVGP
Chinese softshell turtle (*Pelodiscus sinensis*)	UP K7GGY5		QWVIGP
Chinese alligator (*Alligator sinensis*)	UP A0A1U7RBY2		QWVVGP
Burmese python (*Python bivittatus*)	GB XP_025031668		QWVVGP
Amphibians			
African clawed frog (*Xenopus laevis*)	UP Q7SYH2		QWVVGP
Western clawed frog (*Xenopus tropicalis*)	GB XP_002937874		QWVVGP
High Himalaya frog (*Nanorana parkerii*)	GB XP_018422589		QWMFGQ
Axolotl (*Ambystoma mexicanum*)	‡		QWVFGP
Bony fishes			
Channel catfish (*Silurus punctatus*)	UP W5UDH5		QWIVGP
Rainbow trout (*Oncorhynchus mykiss*)	GB XP_021460414		QWVVGP
Atlantic salmon (*Salmo salar*)	UP A0A1S3KKB8		QWVVGP
Turbot (*Scophthalmus maximus*)	UP A0A221J5J5		QWVVGP
Three-spined stickleback (*Gasterosteus aculeatus*)	UP G3NQ86		QWVVGP
Nile tilapia (*Oreochromis niloticus*)	UP I3JY83		QWVVGP
Japanese killifish (*Oryzias latipes*)	UP H2MAI0		QWVVWP
Zebrafish (*Danio rerio*)	UP A5D6T5		QWMVGA
Coelacanth (*Latimeria chalumnae*)	UP M3XK68		QWMVGA
Cartilaginous fishes			
Elephant shark (*Callorhincus milii*)	GB XP_007897454		QGMFFE

† With human fetuin-B.

‡ http://www.ambystoma.org/index.php?option=com_content&view=article&id=67:ambystoma-get-contig&catid=40:codepages&Itemid=2&species=2&mcid=contig331328.
