# Supplementary material for: Structure of mammalian plasma fetuin-B and its mechanism of selective metallopeptidase inhibition
Source: IUCrJ. 2019 Feb 28;6(Pt 2):317–30. doi: 10.1107/S2052252519001568 (PMC6400186; doi:10.1107/S2052252519001568)
Supplement: Supplementary file 1 [file m-06-00317-sup1.pdf]

IUCrJ

Volume 6 (2019)

Supporting information for article:

Structure of mammalian plasma fetuin-B and its mechanism of selective metallopeptidase inhibition

Anna Cuppari, Hagen Koerschgen, Dirk Fahrenkamp, Carlo Schmitz, Tibisay Guevara, Konstantin Karmilin, Michael Kuske, Mario Olf, Eileen Dietzel, Irene Yiallouros, Daniele de Sanctis, Theodoros Goulas, Ralf Weiskirchen, Willi Jahnen-Dechent, Julia Floehr, Walter Stoecker, Luca Jovine and F. Xavier Gomis-Rüth

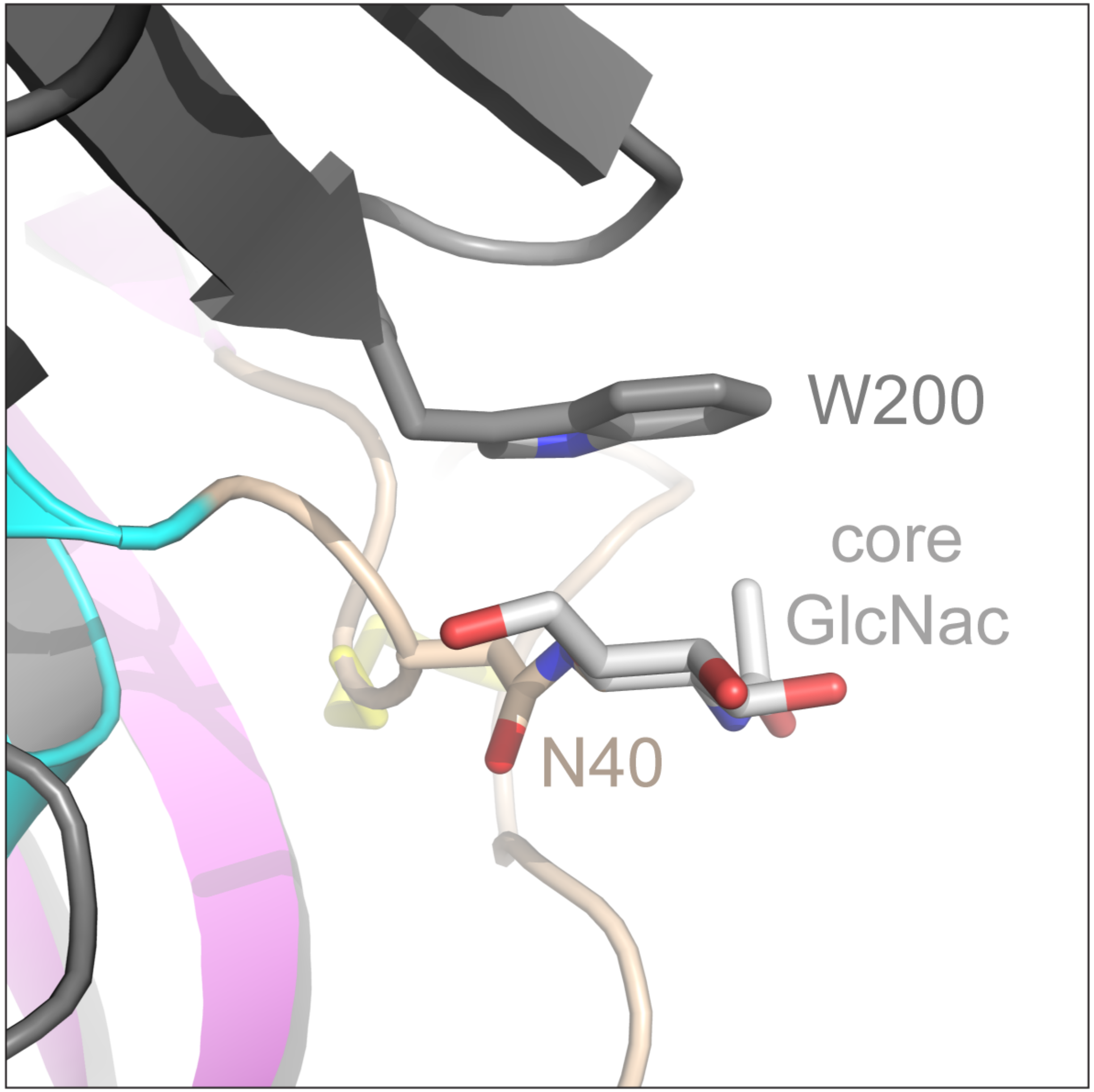

***Supplementary Figure S1* — Crystal packing detail of unbound human fetuin-B.** Examination of the crystal packing of intact mouse fetuin-B from HEK293S cells explains why it crystallized in its unbound form, whereas the material produced in insect cells did not: endoglycosidase H-processing of the high-mannose N-glycans of the HEK293S-derived protein (a step performed during purification) left a single N-acetylglucosamine moiety (GlcNac) attached to N^{40} that stacks against W^{200} of a symmetry-related molecule. This introduces a crucial crystal packing contact that would most likely be hindered by the presence of additional sugar residues attached to the core N-acetylglucosamine, such as those normally found in proteins expressed in insect cells.

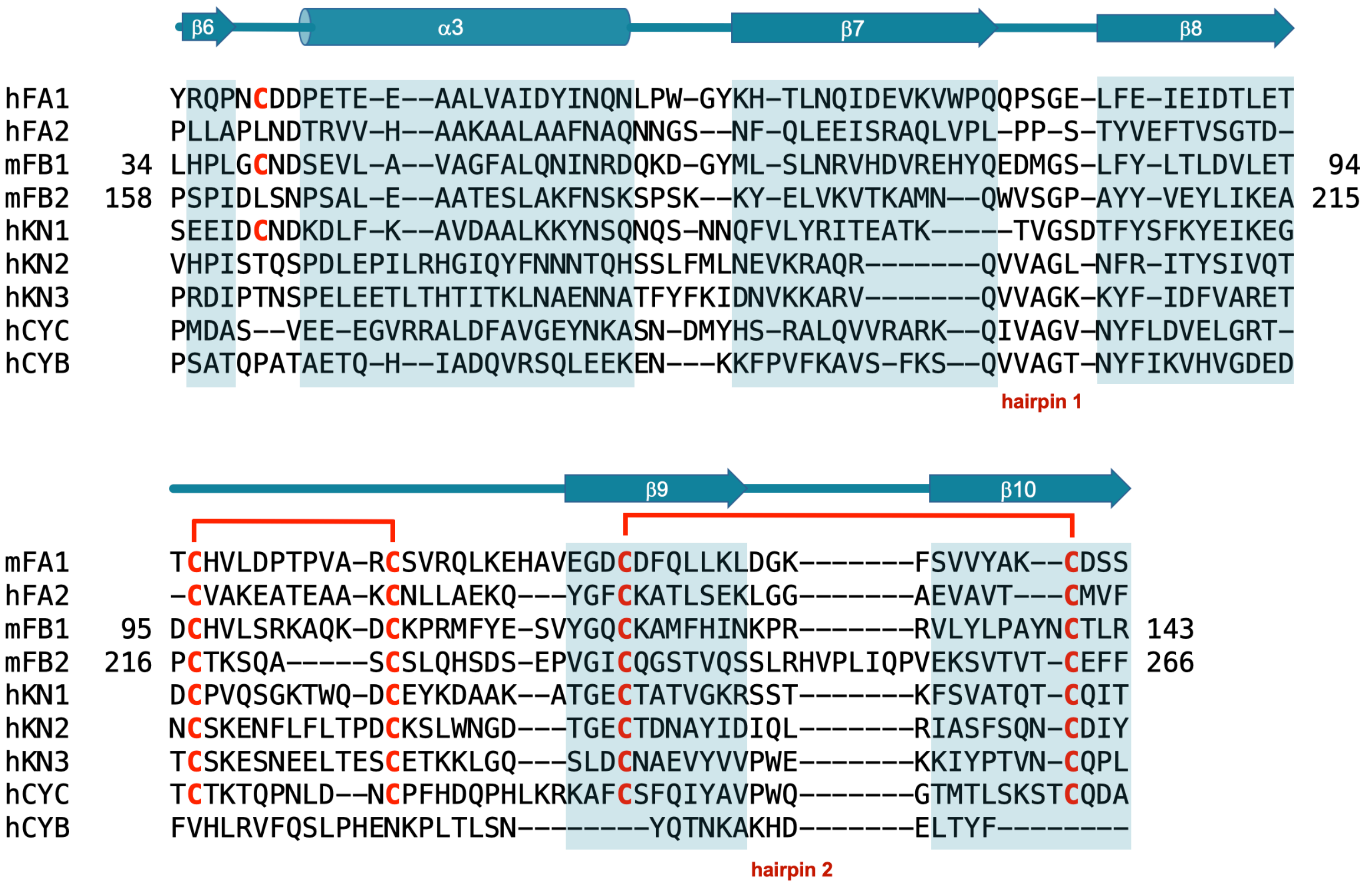

***Supplementary Figure S2* — Sequence alignment of cystatin-like domains.** Alignment of the sequences of cystatin-like modules spanning from the first residue of the first β-strand to the last residue of the last strand, i.e. without N- or C-terminal extensions. hFA1, hFA2: domains 1 and 2 from human fetuin-A (UP P02765); mFA1, mFA2: domains from mouse fetuin-B (UP Q9QXC1); hKN1, hKN2, hKN3: domains from human kininogen-1 (UP P01042); hCYC: human cystatin-C (UP P01034); and hCYB: human cystatin-B *alias* stefin B (UP P04080). The residue numbers correspond to the sequence of mouse fetuin-B. Secondary-structure elements above the alignment are for the CY2 domain of mouse fetuin-B.

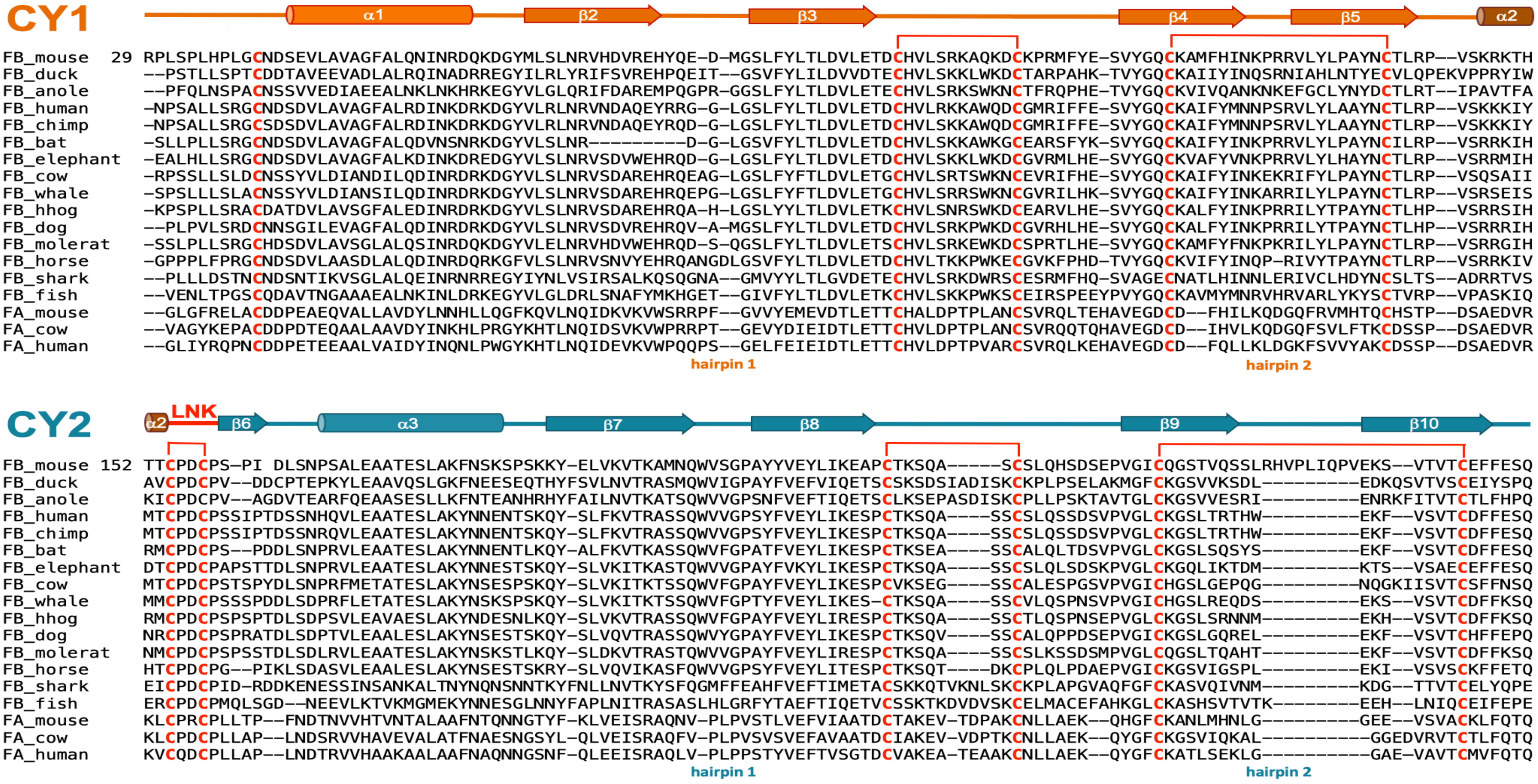

***Supplemental Figure S3* — Amino-acid sequence alignment of selected vertebrate fetuins**. FB_mouse: fetuin-B from *Mus musculus* (UP Q9QXC1); FB-duck: *Anas platyrhynchos* (UP U3IFE7); DB_anole: *Anolis carolinensis* (UP H9GD35); FB_human: *Homo sapiens* (UP Q9UGM5); FB_chimp: *Pan troglodytes* (UP A0A2J8M5X2); FB_bat: *Pteropus alecto* (UP L5JPD4); FB_elephant : *Loxodonta africana* (UP G3SVW6); FB_cow: *Bos taurus* (UP Q58D62); FB_whale; *Physeter catodon* (UP A0A2Y9F7I5); FB_hhog: *Erinaceus europaeus* (UP A0A1S2ZIC8); FB_dog: *Canis familiaris* (UP E2R9B6); FB_molerat: *Heterocephalus glaber* (UP G5BT88); FB_horse: *Equus caballus* (UP F6RRV1); FB_shark: *Callorhinchus milii* (UP V9KTH3); FB_fish: *Danio rerio* (UP E7FE90); FA_mouse: fetuin-A from *Mus musculus* (UP P29699); FA_coe; *Bos taurus* (UP P12763); and FA_human: *Homo sapiens sapiens* (UP P02765). Residue numbers and secondary structure elements correspond to mouse fetuin-B (UP Q9QXC1), see also Fig. 1A. Cysteine residues are in red, the first one (C^{39} in mouse fetuin-B) is linked to a cysteine in the CTR (C^{374}). LNK indicates the localization of the CPDC-trunk in the linker between CY1 and CY2. The hairpin-loops of each domain are indicated.
